# Suppression of Metastatic Ovarian Cancer Cells by Bepridil, a Calcium Channel Blocker

**DOI:** 10.3390/life13071607

**Published:** 2023-07-22

**Authors:** Songzi Zhang, Dokyeong Kim, Minyoung Park, Jing Hu Yin, Junseong Park, Yeun-Jun Chung

**Affiliations:** 1 Precision Medicine Research Center, College of Medicine, The Catholic University of Korea, Seoul 06591, Republic of Korea; 2 Department of Microbiology, College of Medicine, The Catholic University of Korea, Seoul 06591, Republic of Korea; 3 Department of Biomedicine and Health Sciences, College of Medicine, The Catholic University of Korea, Seoul 06591, Republic of Korea

**Keywords:** anticancer therapy, bepridil, epithelial–mesenchymal transition, metastasis, advanced ovarian cancer

## Abstract

Although surgery followed by platinum-based therapy is effective as a standard treatment in the early stages of ovarian cancer, the majority of cases are diagnosed at advanced stages, leading to poor prognosis. Thus, the identification of novel therapeutic drugs is needed. In this study, we assessed the effectiveness of bepridil—a calcium channel blocker—in ovarian cancer cells using two cell lines: SKOV-3, and SKOV-3-13 (a highly metastatic clone of SKOV-3). Treatment of these cell lines with bepridil significantly reduced cell viability, migration, and invasion. Notably, SKOV-3-13 was more sensitive to bepridil than SKOV-3. The TGF-β1-induced epithelial–mesenchymal transition (EMT)-like phenotype was reversed by treatment with bepridil in both cell lines. Consistently, expression levels of EMT-related markers, including vimentin, β-catenin, and Snail, were also substantially decreased by the treatment with bepridil. An in vivo mouse xenograft model was used to confirm these findings. Tumor growth was significantly reduced by bepridil treatment in SKOV-3-13-inoculated mice, and immunohistochemistry showed consistently decreased expression of EMT-related markers. Our findings are the first to report anticancer effects of bepridil in ovarian cancer, and they suggest that bepridil holds significant promise as an effective therapeutic agent for targeting metastatic ovarian cancer.

## 1. Introduction

Ovarian cancer is among the three most prevalent malignancies affecting the female reproductive system, with a mortality rate exceeding 50% [1,2]. The current standard of care for early-stage cases involves cytoreductive surgery followed by platinum-based chemotherapy, achieving an approximate efficacy of 90% [3,4]. However, most ovarian cancer patients are typically diagnosed at advanced stages, characterized by the metastatic dissemination of malignant cells to distant organs, consequently resulting in 5-year survival rates of less than 30% [5,6]. To develop efficacious therapies for high-grade ovarian cancer, it is crucial to investigate the molecular drivers of ovarian cancer’s aggressiveness and identify therapeutic agents to target metastasis.

Bepridil is known as a calcium channel blocker, possessing a distinct electrophysiological profile [7,8]. It is widely utilized as a therapeutic agent for cardiovascular disorders, known for its ability to decrease myocardial oxygen consumption and enhance coronary blood flow [9]. The equilibrium column-binding technique revealed that bepridil exhibits specific binding affinity towards calmodulin—an intracellular calcium receptor protein—in the presence of calcium ions. This binding event consequently leads to the inhibition of calcium ion influx, thereby exerting its calcium-channel-blocking effect [10]. In addition to being an inhibitor of calcium channels, several studies have shown that bepridil can effectively block other ion channels, including the potassium and sodium channels [11,12], indicating its multitarget capacity. In the context of cancer, there have been limited studies investigating the anticancer properties of bepridil, which have demonstrated its potential to promote apoptosis and induce cell-cycle arrest in cancer cells [13,14,15,16]. However, there is currently limited knowledge regarding the specific effects of bepridil on ovarian cancer.

Here, we utilized two human ovarian cancer cell lines, namely, SKOV-3 and SKOV-3-13. SKOV-3-13 is a highly aggressive clone derived from SKOV-3 through repetitive in vivo selection of metastatic cells targeting the peritoneum. This selection process has led to an enhanced capacity for metastasis and an accelerated growth rate [17]. In our prior investigation, we identified the upregulation of the troponin C type 1 (*TNNC1*) gene in SKOV-3-13 compared to SKOV-3 [18]. *TNNC1* is a component of the calcium-regulatory complexes and has been implicated in driving the aggressive behaviors of ovarian cancer [19]. Building on our previous findings, the present study aimed to further explore the potential of bepridil as a targeted therapeutic agent for the modulation of *TNNC1* signaling and the suppression of the metastatic phenotype of ovarian cancer cells.

## 2. Materials and Methods

### 2.1. Cell Culture and Culture Condition

The SKOV-3 human ovarian cancer cell line (American Type Culture Collection, Manassas, VA, USA) and its highly aggressive counterpart SKOV-3-13 (Sunnybrook Health Sciences Centre, Toronto, ON, Canada) [17], WM239A (human melanoma cancer cell line), HepG2 (human hepatocellular cancer cell line), and MCF-7 (human breast cancer cell line) were cultured in RPMI-1640 medium (HyClone, Logan, UT, USA). PC-3 human prostatic adenocarcinoma cells were cultured in DMEM medium containing 4500 mg/L D-glucose (Welgene, Gyeongsan, Republic of Korea). The HEK-293 normal human kidney cell line was cultured in EMEM medium (Quality Biological, Gaithersburg, MD, USA). All cell culture media were supplemented with 10% fetal bovine serum (HyClone) and 1% penicillin–streptomycin (Sigma-Aldrich, Darmstadt, Germany). The cells were maintained in a controlled environment with 5% CO_2_ at 37 °C. Both cell lines were utilized at passages 5–10 for the experiments. The cells were initially placed at a density of 1 × 10^6^ cells per 100 mm dish (SPL, Pocheon, Republic of Korea). The medium was refreshed every 2 days. To induce an epithelial–mesenchymal transition (EMT)-like phenotype, the cells were treated with 10 ng/mL of TGF-β1 (R&D Systems, Minneapolis, MN, USA).

### 2.2. Cell Cytotoxicity Assay

**Figure 1:**
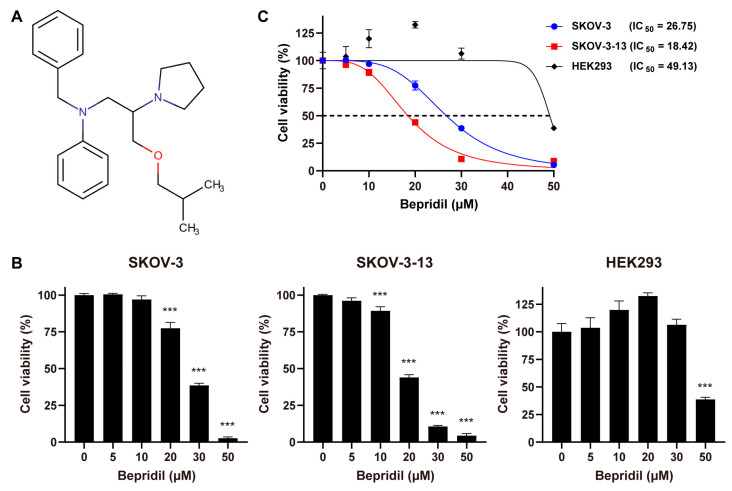
The effects of bepridil on the viability of ovarian cancer cells: (**A**) The chemical structure of bepridil, obtained from the DrugBank database. (**B**,**C**) The cell viability of SKOV-3, SKOV-3-13, and HEK293 cells was measured using WST assays following a 72-h treatment with different concentrations of bepridil. The results are presented in bar graphs (**B**) and curve graphs (**C**). The IC_50_ values were calculated through nonlinear regression analysis of the WST assay results at 72 h post-treatment with bepridil. Statistical significance (one-way ANOVA with Dunnett’s post hoc test) was employed, and the results were reported as the means ± SEM (*** *p* < 0.001). The asterisks above the treatment groups indicate significant statistical differences when compared to the untreated controls.

WST assays with the Cell Counting Kit-8 (Dojindo Molecular Technologies, Kumamoto, Japan) were performed to assess the viability of cells in response to bepridil (Figure 1A; Sigma-Aldrich) or cisplatin (Tocris bioscience, Bristol, UK). Cells were seeded into 96-well plates with 6 × 10^3^ cells per well. Following treatment with gradient concentrations of bepridil or cisplatin, the plates were incubated for 48 or 72 h. A positive control group was included, where the cells were treated with 0.2% triton for 1 h to induce 100% cell death. Following this, 10% of the CCK-8 solution was added compared to the volume of the medium, and the cells were further incubated for an additional 1.5 h. We used a microplate reader (BioTek Instruments Inc., Santa Clara, CA, USA) to measure the absorbance at 450 nm. To compensate for cell viability, the OD values of all experimental wells were extracted equally to those obtained from a positive control group. The 50% inhibitory concentration (IC_50_) was determined through nonlinear regression of the WST results using Prism GraphPad (GraphPad Software, Boston, MA, USA).

### 2.3. Wound-Closure Assay

For the scratch wound-closure assay, both cell lines were seeded into six-well plates (SPL) and cultured until the cell density reached approximately 90%. Then, the bottom of each well was scratched perpendicular to the horizontal line behind the gun head using 200 μL pipette tips (Starlab, Hamburg, Germany). The scratched cells were removed by carefully washing the cells with PBS (HyClone), and the remaining cells were incubated with serum-free medium. The results were derived from bright-field images acquired at the time points of 0, 24, and 48 h. The wound closure was calculated using the ImageJ program.

### 2.4. Transwell Invasion Assay

Eight-micrometer Millicell inserts (SPL) coated with Matrigel (Corning, New York, NY, USA) and serum-free RPMI medium (1:4) were placed into 24-well plates (SPL). In the upper chamber, 100 μL of serum-free medium containing 2 × 10^4^ cells was added, while the lower chamber was filled with 500 μL of serum-containing medium (10% FBS). Subsequently, the plates were incubated for 24 h to facilitate the cells’ attachment to the bottom of the chambers. Following this, bepridil was introduced into the upper chamber. After a further incubation period of 48 h, the non-invading cells within the chamber were meticulously eliminated. The cells embedded in the lower chamber were defined as invaded cells. These cells were fixed with 4% paraformaldehyde and stained using 0.5% crystal violet (Sigma-Aldrich). The membrane image was captured using an upright microscope (Carl Zeiss, Oberkochen, Germany), and the cells were counted in at least three randomly selected fields at 20× magnification per chamber.

### 2.5. Western Blot Analysis

The total proteins were solubilized using an ice-cold RIPA lysis buffer (Biosesang, Seongnam, Republic of Korea), which included a protease inhibitor (GenDEPOT, Katy, TX, USA) and a phosphatase inhibitor cocktail (GenDEPOT). The protein levels were quantified utilizing bovine serum albumin as the standard (BIO-RAD, Hercules, CA, USA). The protein samples (20 μg) were loaded into SDS-PAGE and then transferred to PVDF membranes (Millipore, Billerica, MA, USA). In order to minimize background staining, the membranes were incubated with 5% non-fat dry milk for 1.5 h. Following that, they were immunoblotted overnight at 4 °C using the following antibodies: rabbit anti-β-Actin (1:1000, Cell Signaling Technology, Boston, MA, USA), rabbit anti-vimentin (1:1000, Cell Signaling Technology), rabbit anti-β-catenin (1:1000, Cell Signaling Technology), and rabbit anti-Snail (1:1000, Cell Signaling Technology). Following the washing of the membranes with TBST, they were subsequently incubated with anti-rabbit IgG-HRP (1:4000, Santa Cruz Biotechnology, Dallas, TX, USA) for 1 h at 37 °C. Subsequently, the membranes were exposed to a chemiluminescence substrate (Pico PLUS or Femto Maximum Sensitivity Substrate, Thermo Scientific, Waltham, MA, USA). The signals from the bands were quantified using an Amersham Imager 600 (Fujifilm, Tokyo, Japan), and the results were shown as a relative value to the control set, with a value of 1.0.

### 2.6. In Vivo Mouse Xenograft Models

Five-week-old female healthy BALB/c nude mice weighing 15–20 g were procured from Orient Bio Laboratories (Seoul, Republic of Korea). The mice were housed in a facility with a 12 h light–dark cycle and provided with standard laboratory conditions, including appropriate temperature, humidity, and ventilation. Following a one-week adaptation period, all 20 mice were randomly divided into four groups to examine in vivo tumor growth: SKOV-3-Vehicle, SKOV-3-Bepridil, SKOV-3-13-Vehicle, and SKOV-3-13-Bepridil. Each mouse was subcutaneously injected with the corresponding SKOV-3 or SKOV-3-13 cells (a total of 3 × 10^4^ cells per mouse, mixed with Matrigel/serum-free RPMI at a 1:2 ratio) into the right flank. Once the tumors became visible to the naked eye, the mice were administered intraperitoneally with 20 mg/kg of bepridil, while the vehicle group mice were injected with an equivalent volume of DMSO instead of bepridil. Both groups of mice were injected three times per week, and the in vivo dose of bepridil was determined based on previous studies [20,21]. The tumor sizes were monitored by measuring the tumor diameters once per week. The tumor volume (V) was measured by caliper using the formula V = 1/2 × length × width × width (mm^3^). After five weeks, all mice were euthanatized by CO_2_ inhalation without experiencing pain, and the tumors were dissected and collected for further research. The animal procedures described in this study were conducted following the rules and approved by the institutional animal cancer and use committee of the Catholic University of Korea (CUMS-2021-0352-02).

### 2.7. Tissue Processing and Immunohistochemistry (IHC)

The fresh tumor tissues were fixed in 4% paraformaldehyde for a minimum of 24 h. Subsequently, they were embedded in paraffin, sectioned, and subjected to staining. The tissue sections (4 μm) were deparaffinized using xylene (Duksan Reagents, Ansan, Republic of Korea) and rehydrated using a graded series of ethanol dilutions (Sigma-Aldrich). Then, hematoxylin (Abcam, Cambridge, UK) and eosin (Abcam) were applied to the tissue sections for 20 s. Image acquisition was performed using the Pannoramic SCAN II (3DHISTECH Ltd., Budapest, Hungary).

For IHC, the tissue sections were processed in a similar manner as the hematoxylin staining described above. Specific antibodies were used as follows: vimentin (1:100, Abcam), Snail (1:100, Cell Signaling Technology), and β-catenin (1:100, Cell Signaling Technology). The samples were incubated with the corresponding secondary antibody (Dako Agilent, Santa Clara, CA, USA). After washing with TBST (Biosesang), the specimens were treated in DAB solution for 1–10 min and stained with hematoxylin for a further 20 s. Images were captured using the Pannoramic SCAN II, with at least three randomly selected fields captured per tissue.

### 2.8. Quantification and Statistical Analysis

The data were statistically analyzed using the GraphPad Prism 9.0.0 software (GraphPad Software). The specific statistical methods employed for each analysis are described in the respective figure legends. In brief, comparisons between two groups were assessed using Student’s *t*-test. To compare the multiple concentrations of bepridil, one-way ANOVA was conducted with Dunnett’s post hoc test. The comparisons of tumor sizes between groups at each timepoint were performed using multiple *t*-tests. Statistical significance was defined as a probability of less than 5% (* *p* < 0.05).

## 3. Results

### 3.1. Bepridil Inhibits Ovarian Cancer Cell Proliferation

**Figure 2:**
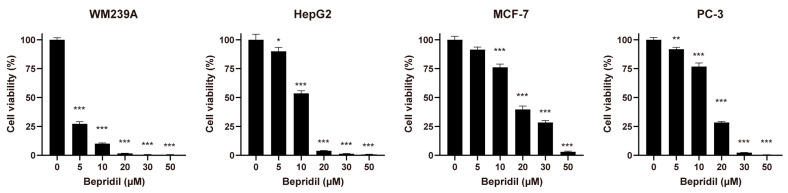
The effects of bepridil on the viability of diverse types of cancer cells: The cell viability of WM239A, HepG2, MCF-7, and PC-3 cells was measured using WST assays after a 72-h treatment with different concentrations of bepridil. Statistical significance was determined using one-way ANOVA with Dunnett’s post hoc test, and the results were depicted as means ± SEM (* *p* < 0.05, ** *p* < 0.01, and *** *p* < 0.001 compared to the untreated controls).

We first assessed the impact of bepridil (Figure 1A) on the cell viability in two ovarian cancer cell lines—namely, SKOV-3 and its metastatic counterpart SKOV-3-13—and a normal cell line (HEK293). Following a 72 h treatment period, bepridil demonstrated a dose-dependent reduction in cell viability for both ovarian cancer cell lines. However, there was a significant contrast observed in HEK293 cells, which displayed notable resistance to bepridil compared to ovarian cancer cells. HEK293 cells exhibited an increase in viability upon treatment with bepridil at concentrations lower than 30 µM (Figure 1B,C). These findings strongly suggest the cancer-specificity of bepridil. Importantly, the IC_50_ value for SKOV-3-13 cells was lower than that for SKOV-3 cells at the 72 h timepoint (Figure 1C), indicating a greater sensitivity of SKOV-3-13 cells to bepridil compared with SKOV-3 cells. In contrast, treatment with cisplatin as a reference agent, which is a widely used chemotherapeutic drug for ovarian cancer, resulted in comparable cytotoxicity across all tested cells, indicating a lack of selectivity (Figure S1). To ensure that sublethal concentrations of bepridil were utilized for subsequent functional assays, we selected a concentration of 15 µM, which falls below the IC_50_ values of both ovarian cancer cell lines (Figure 1C), for further in vitro experiments. Furthermore, we investigated the anticancer efficacy of bepridil against melanoma, hepatocellular carcinoma, breast cancer, and prostate cancer cells (Figure 2). Bepridil demonstrated significant anticancer efficacy against all tested cancer cells, with particularly noteworthy effects observed in WM239A and HepG2 cells. These findings suggest the potential for bepridil to be applied to various types of cancer.

### 3.2. Bepridil Inhibits Ovarian Cancer Cell Migration and Invasion, with Reduced Expression of EMT-Associated Genes

**Figure 3:**
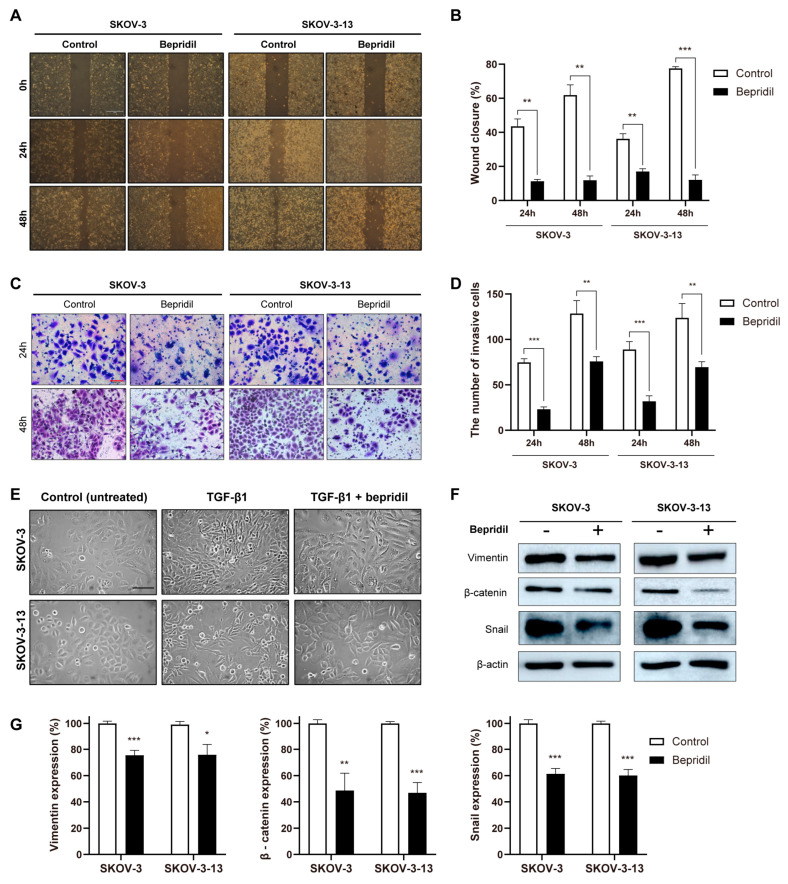
Inhibition of cell migration and invasion by bepridil in ovarian cancer cells: (**A**) Representative images from wound-healing assays at 24 and 48 h were captured for both the untreated control group and the bepridil-treated (15 μM) group. The scale bar represents 1 mm. (**B**) Bar graph indicating the percentage of wound closure. (**C**) Transwell invasion assays of both cell lines in the untreated control and bepridil-treated (15 μM) groups at 24 and 48 h. The scale bar in the images represents 100 μm. (**D**) Bar graph illustrating the total number of invasive cells in each group. (**E**) Bright-field images demonstrating the alterations in the cellular morphology of SKOV-3 and SKOV-3-13 cells following a 48-h treatment with bepridil (15 μM) and TGF-β1 (10 ng/mL). (**F**) Representative Western blot images displaying the expression of vimentin, β-catenin, and Snail in the untreated control (−) and bepridil-treated (+) groups of SKOV-3 and SKOV-3-13 cells. β-Actin was employed as a loading control to ensure equal protein loading across the samples. (**G**) The quantifications of band intensities in the untreated control and bepridil-treated (15 μM) groups of both cell lines using the ImageJ program. For (**B**,**D**,**G**), the mean values with SEMs were calculated, and statistical analysis (Student’s *t*-test) was performed, with significance levels indicated as * *p* < 0.05, ** *p* < 0.01, and *** *p* < 0.001. Asterisks positioned above the treatment groups indicate significant differences when compared to the untreated controls.

To assess the potential of bepridil in inhibiting migration and invasion in ovarian cancer cells, we conducted wound-closure assays and transwell migration and invasion assays. At 24 and 48 h after creating the scratch, both SKOV-3 and SKOV-3-13 cells demonstrated migration into the wound area, while treatment with bepridil significantly reduced cell migration (Figure 3A,B). Consistent results were obtained from the transwell migration assays (Figure S2), confirming that bepridil inhibits the migratory phenotype of ovarian cancer cells. Additionally, transwell invasion assays showed that treatment with bepridil for 24 and 48 h significantly decreased the number of invaded cells in both SKOV-3 and SKOV-3-13 cells (Figure 3C,D).

To provide a mechanistic explanation underlying the inhibitory effects of bepridil on migration and invasion, we focused on the EMT, a well-known process associated with cancer cell migration and invasion. We induced an EMT-like phenotype in both cell lines using TGF-β1, a known inducer of EMT [22], and observed that bepridil treatment reversed this morphological change (Figure 3E). Furthermore, we evaluated the protein levels of EMT-related markers, including β-catenin, Snail, and vimentin, through Western blot analysis. Bepridil treatment significantly downregulated the expression of these proteins in both cell lines (Figure 3F,G). These findings collectively suggest that bepridil suppresses the migratory and invasive phenotypes of ovarian cancer cells, accompanied by reduced expression of EMT-associated genes.

### 3.3. Bepridil Suppresses Tumor Growth in a Mouse Ovarian Cancer Xenograft Model

**Figure 4:**
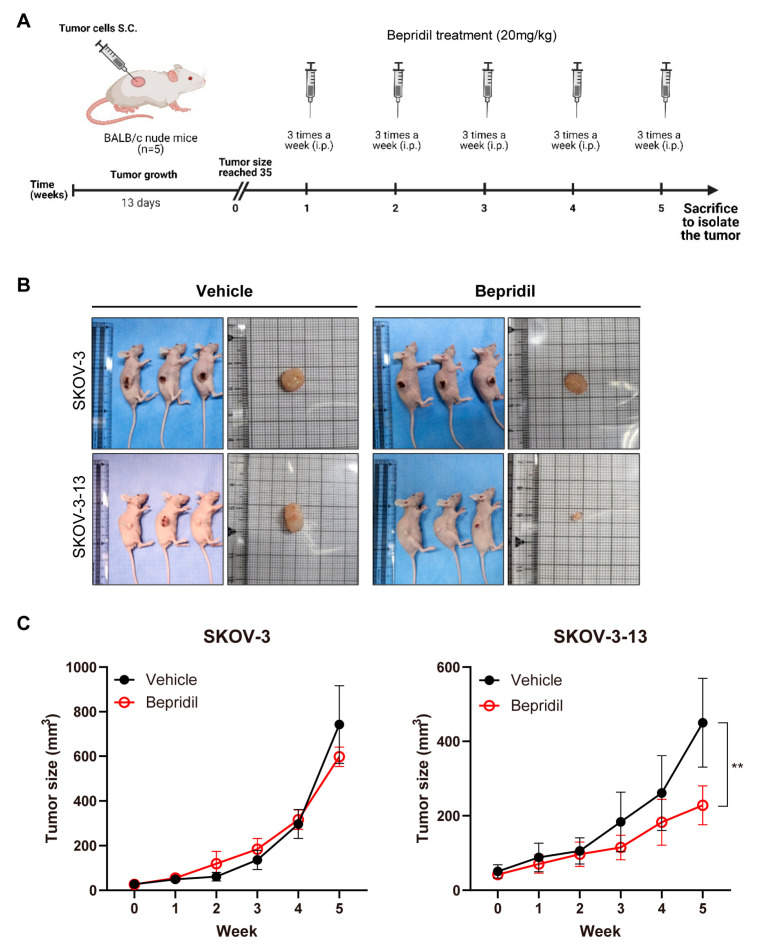
The effects of bepridil on the in vivo tumor growth of ovarian cancer cells: (**A**) The treatment schedule of the in vivo experiments. (**B**) Representative images of euthanatized mice and tumors obtained from each group were captured. Five-week-old female BALB/c nude mice were subcutaneously inoculated with SKOV-3 and SKOV-3-13 cells. Once the tumor size became visible, the mice were treated with either 22 mg/kg bepridil or the same volume of DMSO as a vehicle (*n* = 5 per group). (**C**) The quantified tumor sizes of each group. The mean values with SEMs were calculated, and statistical analysis (multiple *t*-tests) was performed, with significance levels denoted as ** *p* < 0.01. The differences between groups are indicated for the last timepoint.

To evaluate the efficacy of bepridil in vivo, we established a mouse xenograft model by subcutaneously injecting SKOV-3 and SKOV-3-13 cells. Once the tumors became measurable (13 days after inoculation), the mice were treated intraperitoneally with bepridil three times per week for a duration of five weeks (Figure 4A). Importantly, the bepridil treatment led to a notable decrease in the tumor size compared to the control group, without observable systemic toxicity (Figure S3), specifically in the SKOV-3-13 model and not in the SKOV-3 model (Figure 4B,C). These findings were consistent with the results obtained from the in vitro cell viability assays, which showed that SKOV-3-13 cells were more sensitive to the effects of bepridil (Figure 1).

**Figure 5:**
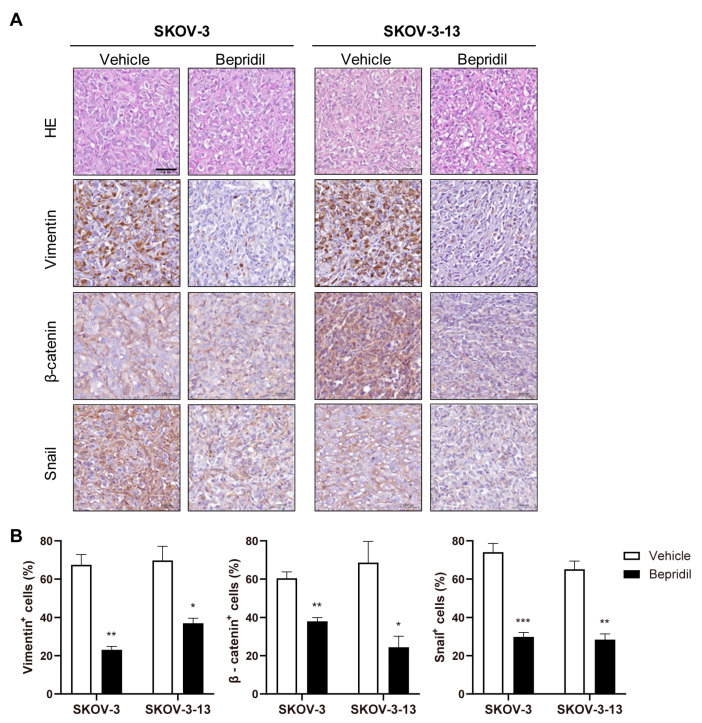
Downregulation of EMT-related marker proteins by bepridil in mouse xenograft models: (**A**) Representative IHC images of H&E staining, vimentin, β-catenin, and Snail. The scale bar represents 50 µm, and the magnification is 400×. (**B**) Bar graphs were generated to illustrate the percentage of stained cells, indicating the proportion of marker-positive cells. The mean values with SEMs were calculated, and statistical analysis (Student’s *t*-test) was performed, with significance levels indicated as * *p* < 0.05, ** *p* < 0.01, and *** *p* < 0.001. Asterisks positioned above the treatment groups indicate significant differences when compared to the vehicle controls.

We also performed IHC analysis to quantify the expression of EMT marker proteins in tumor tissues obtained from xenograft mice that were euthanatized at the same timepoint (5 weeks after inoculation). Immunostaining revealed that treatment with bepridil significantly reduced the expression of β-catenin, Snail, and vimentin in both the SKOV-3 and SKOV-3-13 groups (Figure 5A,B). These findings are consistent with the observed morphological changes and the results obtained from the Western blot analysis (Figure 3E–G). Collectively, these findings demonstrate that bepridil exhibits in vivo efficacy in ovarian cancer cells by downregulating EMT-related genes, with enhanced therapeutic responses observed in mice inoculated with the metastatic clone SKOV-3-13.

## 4. Discussion

Ovarian cancer is frequently characterized by high rates of recurrence and acquired resistance to chemotherapy, leading to unfavorable overall survival outcomes [23,24]. Consequently, the development of novel therapeutic agents for advanced ovarian cancer is of utmost importance. Bepridil, a non-selective ion channel blocker approved by the FDA, is widely employed in the treatment of arrhythmia and heart failure [9,25]. Its pharmacological actions include reducing calcium influx through receptor-operated and voltage-dependent calcium channels, acting as a calmodulin antagonist and intracellular calcium sensitizer [9,26]. Moreover, bepridil has demonstrated potential efficacy in combating viral diseases, such as its considerable anti-SARS-CoV-2 activity, exhibiting dose-dependent effectiveness in A459/ACE2 and Vero E6 cells [25]. Beyond its calcium-ion-inhibition properties, bepridil also possesses the ability to block potassium ion efflux [27]. Recent studies have suggested that antagonism of ATP-sensitive potassium channels holds promise as a therapeutic strategy for various cancers and neurodegenerative disorders [28,29]. This opens up a new research direction for bepridil as a treatment option. However, despite its potential, the antitumor effects of bepridil on ovarian cancer remain largely unknown. Hence, the objective of this study was to examine the effectiveness of bepridil and provide a mechanistic explanation underlying its action in ovarian cancer cells.

Metastasis is the primary cause of mortality in patients with ovarian cancer [6,30]. Utilizing metastasis models can aid in the identification of specific targets and agents that may hinder or potentially reverse cancer growth and metastasis. Therefore, we selected the SKOV-3 and SKOV-3-13 models, which represent primary and metastatic ovarian cancer, respectively. SKOV-3-13 is a metastatic clone of ovarian cancer derived from SKOV-3 and exhibits a high level of peritoneal dissemination and carcinomatosis in a mouse xenograft model [17]. Our previous research demonstrated significantly elevated *TNNC1* expression in SKOV-3-13 compared to SKOV-3, highlighting the pivotal role of *TNNC1* in accelerating the malignant progression of ovarian cancer cells through the activation of EMT [18]. Corroborating our previous work, this study demonstrates the effectiveness of bepridil—a pharmacological inhibitor of *TNNC1*—in both ovarian cancer cell lines. However, our focus in this study was not on the functional alterations of *TNNC1* and calcium channels, as our primary objective was to evaluate the anticancer efficacy of bepridil and the changes in cancer-related phenotypes. Furthermore, bepridil is not a targeted therapy agent with a specific single target. Instead, it may have multiple targets, including calcium-channel- and potassium-channel-associated genes, as well as several unknown genes [10,11]. This is why our focus was on phenotypic changes and in vivo efficacy rather than specific target inhibition. Since we demonstrated that bepridil suppressed the expression of EMT-associated genes and subsequent morphological changes (Figure 3E–G and Figure 5), EMT- or metastasis-involved genes could be additional targets of bepridil. Some studies have shown that bepridil has apoptotic effects in melanoma, glioblastoma, and leukemia cells [13,14,15,16]. The results of our study demonstrate that bepridil exhibited similar effects on ovarian cancer cells both in vivo and in vitro. Apart from its influence on cell viability, bepridil significantly attenuated the migratory and invasive capabilities of ovarian cancer cells, indicating its potential therapeutic application in the context of metastatic ovarian cancer.

EMT has been widely recognized as a critical hallmark of cancer’s development and metastasis due to its ability to enhance the invasive and migratory properties of cancer cells, as well as to confer resistance to apoptotic stimuli. Consequently, targeting of EMT has emerged as a promising strategy for cancer treatment [31]. Transcription factors, such as Snail and β-catenin, play pivotal roles in driving the invasiveness and stemness of cancer cells during the progression of EMT [32]. For instance, Snail- and β-catenin-mediated EMT has been shown to enhance DNA-repair capacity through the activation of poly ADP-ribose polymerase (PARP) [33], as well as to induce chemoresistance by inhibiting p53-mediated apoptosis [34]. In addition, mounting evidence suggests that EMT contributes to the development of resistance to platinum-based chemotherapy in ovarian cancer [3,35,36]. In light of our findings, which demonstrate that bepridil attenuated EMT-like morphological changes and downregulated the expression of vimentin, β-catenin, and Snail, we provide a mechanistic explanation for how bepridil exerts its suppressive effects on ovarian cancer cells, particularly those exhibiting a metastatic phenotype.

In conclusion, our study provides compelling evidence for the effective inhibition of various malignant biological behaviors, including cell proliferation, motility, and invasion, by bepridil in ovarian cancer cells. Importantly, we are the first to report the attenuation of EMT-related molecules—specifically vimentin, β-catenin, and Snail—by bepridil. This unique mechanism of action positions bepridil as a potential candidate for combination therapy with other treatment modalities, including first-line treatments, while avoiding redundancy in mechanism. We believe that bepridil holds great promise as an appealing therapeutic agent for patients with advanced ovarian cancer. Further investigations are warranted to fully develop a clinically relevant therapeutic strategy involving the utilization of bepridil.

## Data Availability

Not applicable.

## Supplementary Materials

The following are available online at https://www.mdpi.com/article/10.3390/life13071607/s1, Figure S1. The effect of cisplatin on the viability of ovarian cancer cells. (A,B) The cell viability of SKOV-3, SKOV-3-13, and HEK293 cells was measured using WST assays following a 72-h treatment with different concentrations of cisplatin. The results are presented in bar graphs (A) and curve graphs with non-linear regression (B). Statistical significance (one-way ANOVA with Dunnett’s post hoc test) was employed, and the results were reported as means ± SEM (*** *p* < 0.001). The asterisks above the treatment groups indicate significant statistical differences when compared to the untreated controls. Figure S2. Transwell migration assays. Transwell migration assays of SKOV-3 and SKOV-3-13 cells in the untreated control and bepridil-treated (15 μM) groups at 48 h. The migratory cells were fixed and stained with 0.5% crystal violet. Scale bar: 100 μm. Figure S3. The effect of bepridil on the body weight of a mouse ovarian cancer xenograft model. Body weight of all mice were measured weakly. The mean values with SEM were calculated, and statistical analysis (multiple *t*-test) was performed. All data points indicate no significant differences between groups.

## References

[1] Sung H., Ferlay J., Siegel R.L., Laversanne M., Soerjomataram I., Jemal A., Bray F. (2021). Global Cancer Statistics 2020: GLOBOCAN Estimates of Incidence and Mortality Worldwide for 36 Cancers in 185 Countries. CA Cancer J. Clin..

[2] Bae H.S., Kim H.J., Hong J.H., Lee J.K., Lee N.W., Song J.Y. (2014). Obesity and epithelial ovarian cancer survival: A systematic review and meta-analysis. J. Ovarian Res..

[3] Yang L., Xie H.J., Li Y.Y., Wang X., Liu X.X., Mai J. (2022). Molecular mechanisms of platinum-based chemotherapy resistance in ovarian cancer (Review). Oncol. Rep..

[4] Pomel C., Jeyarajah A., Oram D., Shepherd J., Milliken D., Dauplat J., Reynolds K. (2007). Cytoreductive surgery in ovarian cancer. Cancer Imaging.

[5] Matulonis U.A., Sood A.K., Fallowfield L., Howitt B.E., Sehouli J., Karlan B.Y. (2016). Ovarian cancer. Nat. Rev. Dis. Primers.

[6] Wang Y., Shan X., Dong H., Li M., Yue Y. (2022). Prediction for 2-year mortality of metastatic ovarian cancer patients based on surveillance, epidemiology, and end results database. Front. Surg..

[7] Singh B.N. (1992). Bepridil therapy: Guidelines for patient selection and monitoring of therapy. Am. J. Cardiol..

[8] Wei Y.L., Lei Y.Q., Ye Z.J., Zhuang X.D., Zhu L.P., Wang X.R., Cao H. (2023). Effects of bepridil on early cardiac development of zebrafish. Cell Tissue Res..

[9] Hollingshead L.M., Faulds D., Fitton A. (1992). Bepridil: A review of its pharmacological properties and therapeutic use in stable angina pectoris. Drugs.

[10] Itoh H., Tanaka T., Mitani Y., Hidaka H. (1986). The binding of the calcium channel blocker, bepridil, to calmodulin. Biochem. Pharmacol..

[11] Li Y., Sato T., Arita M. (1999). Bepridil blunts the shortening of action potential duration caused by metabolic inhibition via blockade of ATP-sensitive K(+) channels and Na(+)-activated K(+) channels. J. Pharmacol. Exp. Ther..

[12] Sato N., Nishimura M., Kawamura Y., Ward C.A., Kikuchi K. (1996). Block of Na+ channel by bepridil in isolated guinea-pig ventricular myocytes. Eur. J. Pharmacol..

[13] Baldoni S., Del Papa B., Dorillo E., Aureli P., De Falco F., Rompietti C., Sorcini D., Varasano E., Cecchini D., Zei T. (2018). Bepridil exhibits anti-leukemic activity associated with NOTCH1 pathway inhibition in chronic lymphocytic leukemia. Int. J. Cancer.

[14] Lee Y.S., Sayeed M.M., Wurster R.D. (1995). Intracellular Ca^2+^ mediates the cytotoxicity induced by bepridil and benzamil in human brain tumor cells. Cancer Lett..

[15] Hu H.J., Wang S.S., Wang Y.X., Liu Y., Feng X.M., Shen Y., Zhu L., Chen H.Z., Song M. (2019). Blockade of the forward Na(+)/Ca(2+) exchanger suppresses the growth of glioblastoma cells through Ca(2+) -mediated cell death. Br. J. Pharmacol..

[16] Liu Z., Cheng Q., Ma X., Song M. (2022). Suppressing Effect of Na(+)/Ca(2+) Exchanger (NCX) Inhibitors on the Growth of Melanoma Cells. Int. J. Mol. Sci..

[17] Hashimoto K., Man S., Xu P., Cruz-Munoz W., Tang T., Kumar R., Kerbel R.S. (2010). Potent preclinical impact of metronomic low-dose oral topotecan combined with the antiangiogenic drug pazopanib for the treatment of ovarian cancer. Mol. Cancer Ther..

[18] Yin J.H., Elumalai P., Kim S.Y., Zhang S.Z., Shin S., Lee M., Chung Y.J. (2021). TNNC1 knockout reverses metastatic potential of ovarian cancer cells by inactivating epithelial-mesenchymal transition and suppressing F-actin polymerization. Biochem. Biophys. Res. Commun..

[19] Leung C.S., Yeung T.L., Yip K.P., Pradeep S., Balasubramanian L., Liu J., Wong K.K., Mangala L.S., Armaiz-Pena G.N., Lopez-Berestein G. (2014). Calcium-dependent FAK/CREB/TNNC1 signalling mediates the effect of stromal MFAP5 on ovarian cancer metastatic potential. Nat. Commun..

[20] Constantin M., Bromont C., Fickat R., Massingham R. (1990). Studies on the activity of bepridil as a scavenger of free radicals. Biochem. Pharmacol..

[21] DeWald L.E., Dyall J., Sword J.M., Torzewski L., Zhou H., Postnikova E., Kollins E., Alexander I., Gross R., Cong Y. (2018). The Calcium Channel Blocker Bepridil Demonstrates Efficacy in the Murine Model of Marburg Virus Disease. J. Infect. Dis..

[22] Bhowmick N.A., Ghiassi M., Bakin A., Aakre M., Lundquist C.A., Engel M.E., Arteaga C.L., Moses H.L. (2001). Transforming growth factor-beta1 mediates epithelial to mesenchymal transdifferentiation through a RhoA-dependent mechanism. Mol. Biol. Cell.

[23] Pokhriyal R., Hariprasad R., Kumar L., Hariprasad G. (2019). Chemotherapy Resistance in Advanced Ovarian Cancer Patients. Biomark. Cancer.

[24] Giornelli G.H. (2016). Management of relapsed ovarian cancer: A review. Springerplus.

[25] Vatansever E.C., Yang K.S., Drelich A.K., Kratch K.C., Cho C.C., Kempaiah K.R., Hsu J.C., Mellott D.M., Xu S., Tseng C.K. (2021). Bepridil is potent against SARS-CoV-2 In Vitro. Proc. Natl. Acad. Sci. USA.

[26] Li Y., Love M.L., Putkey J.A., Cohen C. (2000). Bepridil opens the regulatory N-terminal lobe of cardiac troponin C. Proc. Natl. Acad. Sci. USA.

[27] Ma F., Takanari H., Masuda K., Morishima M., Ono K. (2016). Short- and long-term inhibition of cardiac inward-rectifier potassium channel current by an antiarrhythmic drug bepridil. Heart Vessel..

[28] Maqoud F., Zizzo N., Attimonelli M., Tinelli A., Passantino G., Antonacci M., Ranieri G., Tricarico D. (2023). Immunohistochemical, pharmacovigilance, and omics analyses reveal the involvement of ATP-sensitive K(+) channel subunits in cancers: Role in drug-disease interactions. Front. Pharmacol..

[29] Maqoud F., Scala R., Hoxha M., Zappacosta B., Tricarico D. (2022). ATP-sensitive Potassium Channel Subunits in Neuroinflammation: Novel Drug Targets in Neurodegenerative Disorders. CNS Neurol. Disord. Drug Targets.

[30] Deng K., Yang C., Tan Q., Song W., Lu M., Zhao W., Lou G., Li Z., Li K., Hou Y. (2018). Sites of distant metastases and overall survival in ovarian cancer: A study of 1481 patients. Gynecol. Oncol..

[31] Brabletz T., Kalluri R., Nieto M.A., Weinberg R.A. (2018). EMT in cancer. Nat. Rev. Cancer.

[32] Ribatti D., Tamma R., Annese T. (2020). Epithelial-Mesenchymal Transition in Cancer: A Historical Overview. Transl. Oncol..

[33] Moyret-Lalle C., Prodhomme M.K., Burlet D., Kashiwagi A., Petrilli V., Puisieux A., Seimiya H., Tissier A. (2022). Role of EMT in the DNA damage response, double-strand break repair pathway choice and its implications in cancer treatment. Cancer Sci..

[34] Parfenyev S., Singh A., Fedorova O., Daks A., Kulshreshtha R., Barlev N.A. (2021). Interplay between p53 and non-coding RNAs in the regulation of EMT in breast cancer. Cell Death Dis..

[35] Marchini S., Fruscio R., Clivio L., Beltrame L., Porcu L., Fuso Nerini I., Cavalieri D., Chiorino G., Cattoretti G., Mangioni C. (2013). Resistance to platinum-based chemotherapy is associated with epithelial to mesenchymal transition in epithelial ovarian cancer. Eur. J. Cancer.

[36] Duan X., Luo M., Li J., Shen Z., Xie K. (2022). Overcoming therapeutic resistance to platinum-based drugs by targeting Epithelial-Mesenchymal transition. Front. Oncol..

