# Supplementary material for: Suppression of Metastatic Ovarian Cancer Cells by Bepridil, a Calcium Channel Blocker
Source: Life (Basel). 2023 Jul 22;13(7):1607. doi: 10.3390/life13071607 (PMC10381520; doi:10.3390/life13071607)
Supplement: Supplementary file 1 [file life-13-01607-s001.zip › Supplementary Figures.pdf]

Supplementary Figures

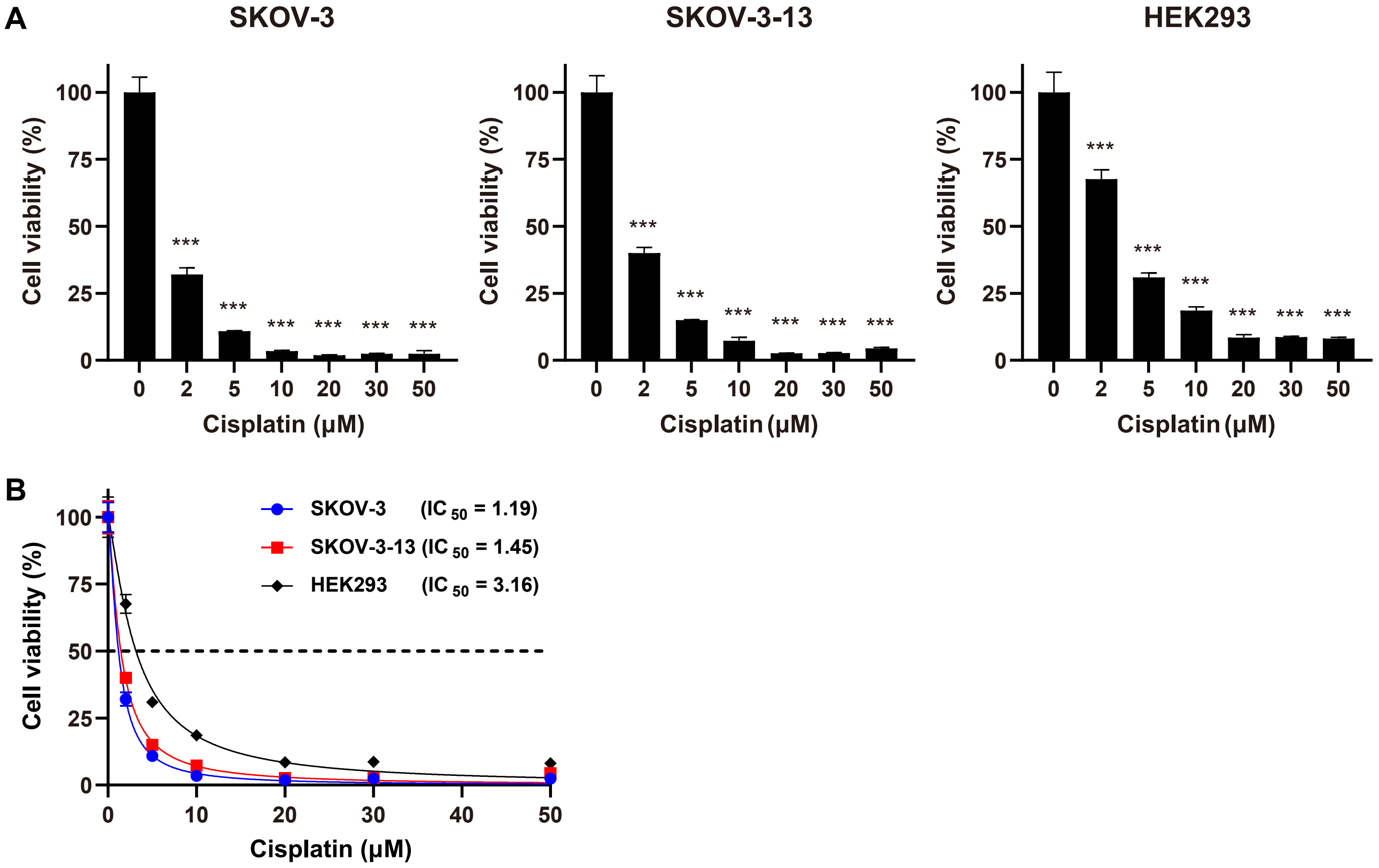

Figure S1. The effect of cisplatin on the viability of ovarian cancer cells. (**A**, **B**) The cell viability of SKOV-3, SKOV-3-13, and HEK293 cells was measured using WST assays following a 72-h treatment with different concentrations of cisplatin. The results are presented in bar graphs (**A**) and curve graphs with non-linear regression (**B**). Statistical significance (one-way ANOVA with Dunnett's *post hoc* test) was employed, and the results were reported as means ± SEM (*** $P < 0.001$). The asterisks above the treatment groups indicate significant statistical differences when compared to the untreated controls.

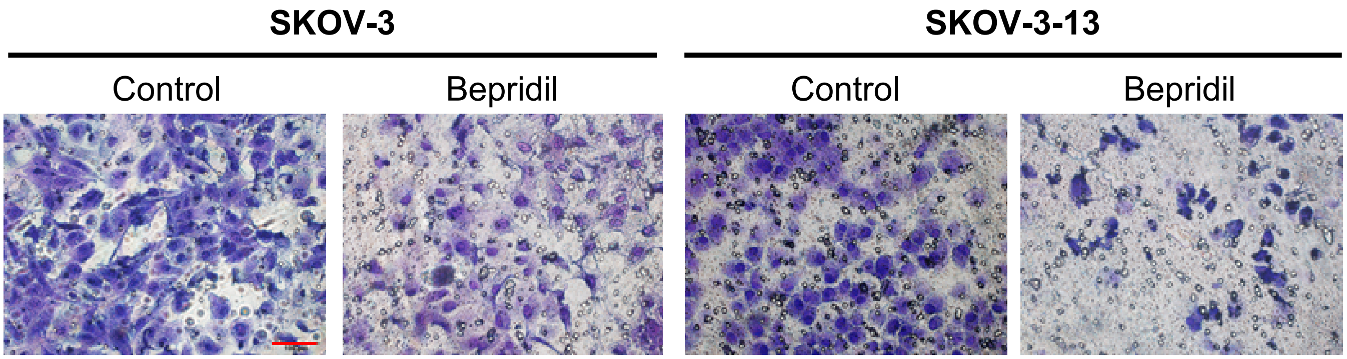

Figure S2. Transwell migration assays. Transwell migration assays of SKOV-3 and SKOV-3-13 cells in the untreated control and bepridil-treated (15 μM) groups at 48 h. The migratory cells were fixed and stained with 0.5% crystal violet. Scale bar: 100 μm.

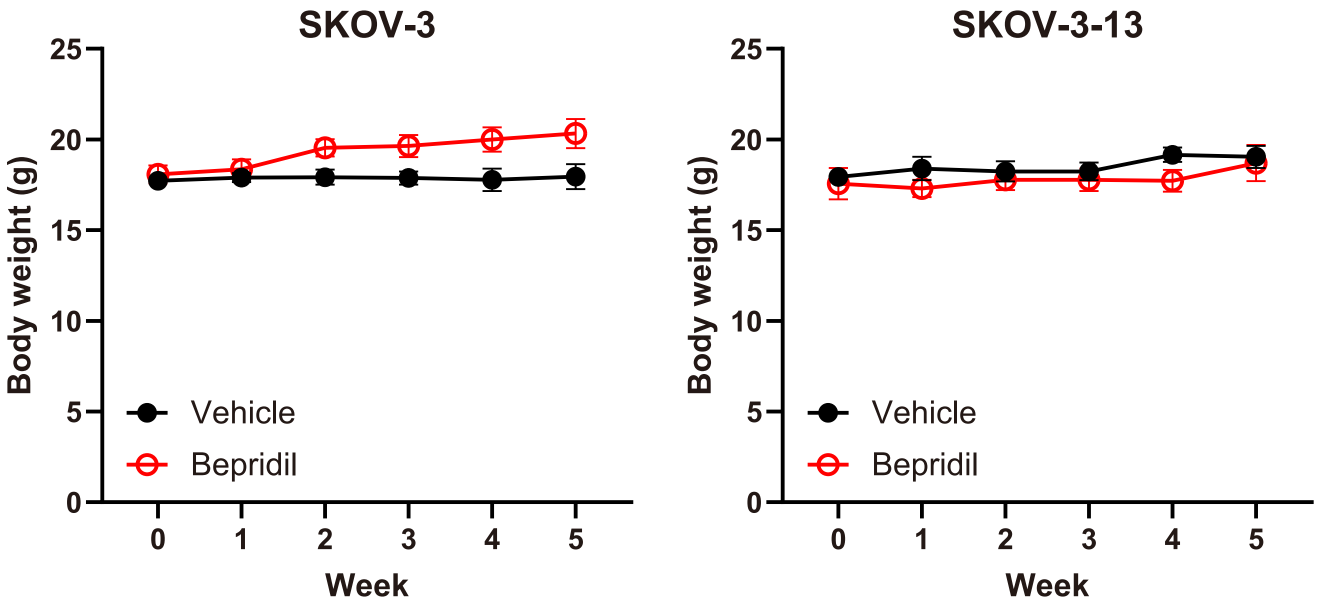

Figure S3. The effect of bepridil on the body weight of a mouse ovarian cancer xenograft model. Body weight of all mice were measured weakly. The mean values with SEM were calculated, and statistical analysis (multiple *t*-test) was performed. All data points indicate no significant differences between groups.
